# A Study of the Pressure-Induced Solidification of Polymers

**DOI:** 10.3390/polym10080847

**Published:** 2018-08-01

**Authors:** Xiuru Liu, Linji Zhang, Chaosheng Yuan, Ru Jia, Chunguang Shao, Mingyou Wang, Shiming Hong

**Affiliations:** School of Physical Science and Technology, Key Laboratory of Advanced Technologies of Materials, Ministry of Education of China, Southwest Jiaotong University, Chengdu 610031, China; zhanglj@hpstar.ac.cn (L.Z.); zzyuancs@163.com (C.Y.); rujia0421@163.com (R.J.); shaochg@zzu.edu.cn (C.S.); 15528237753@163.com (M.W.); smhong2@163.com (S.H.)

**Keywords:** pressure jump, pressure-induced solidification, polymer, amorphous phase, mesomorphic phase

## Abstract

By using a self-designed pressure-jump apparatus, we investigated the melt solidification behavior in the rapid compression process for poly-ethylene-terephthalate (PET), polyether-ether-ketone (PEEK), isotactic polypropylene (iPP), high-density polyethylene (HDPE), and the living polymer sulfur. The experimental results clearly show that crystallization could be inhibited, and some melts were solidified to the full amorphous state for PET, PEEK, and sulfur. Full amorphous PEEK that was 24 mm in diameter and 12 mm in height was prepared, which exceeded the size obtained by the melt quenching method. The bulk amorphous sulfur thus obtained exhibited extraordinarily high thermal stability, and an abnormal exothermic transition to liquid sulfur was observed at around 396 K. Since the solidification of melt is realized by changing pressure instead of temperature and is not essentially limited by thermal conductivity, it is a promising way to prepare fully amorphous polymers. In addition, novel properties are also expected in these polymers solidified by the pressure-jump within milliseconds.

## 1. Introduction

**Figure 1:**
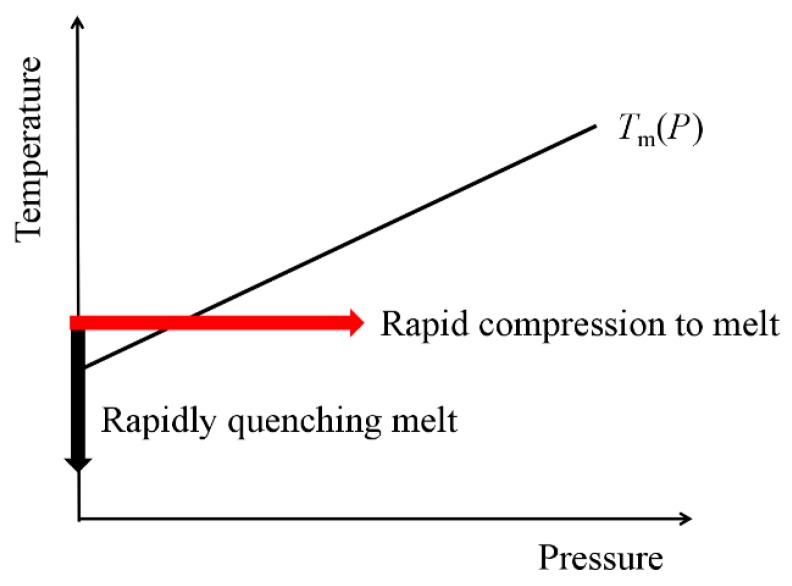
Two possible routes to obtain full amorphous polymers.

Alongside temperature and composition, pressure is another independent parameter that determines the structure, state, and properties of materials. Fully exploiting the pressure variable promises to add a new dimension to the scientific exploration of a broad range of fields, including the polymer materials field [1]. The structure and properties of polymer materials are significantly affected by pressure [2]. The crystallization, isothermal annealing, and melt quenching under high pressure has been studied intensively in polyolefin, polyester, and polyamide [3,4,5]. Most polymer materials are semicrystalline polymers composed of crystalline and amorphous phases. Figure 1 shows two routes to prepare full amorphous polymers. Cooling is the most widespread process for the solidification of melts. It could undergo a melt-to-crystal transition or a melt-to-metastable phase (e.g., amorphous) transition under different cooling rates, which means the rate dependence of solidification [6,7]. Molecular dynamics simulations predict that any melt can be solidified to be a fully amorphous solid state under a cooling rate of 10^12^ K/s [8]. The cooling rates of common quenching methods are around 10^3^–10^7^ K/s [9]. Compression also leads to the solidification of the melts. This process is thermodynamically symmetrical to the cooling process. As shown in Figure 1, for those polymers whose melting temperature increases with increasing pressure, compression should lead to the solidification of their melts. Furthermore, if the increase of pressure is sufficiently high and rapid, the melt should be frozen to an amorphous solid through this non-equilibrium process [10]. To obtain a full amorphous phase, sufficiently deep supercooling and a rapid compressing rate are necessary. That is to say that the pressure of melt should jump to a high value within a very short time. In this process, the solidification of melt is realized by changing the pressure instead of the temperature, so the preparation of the amorphous phase should not be limited by the thermal conductivity. In this work, we reviewed our studies on the pressure-induced solidification of polymer melts, including the poly-ethylene-terephthalate (PET), polyether-ether-ketone (PEEK), isotactic polypropylene (iPP), high-density polyethylene (HDPE), and the living polymer sulfur.

## 2. Experimental Apparatus and Details

**Figure 2:**
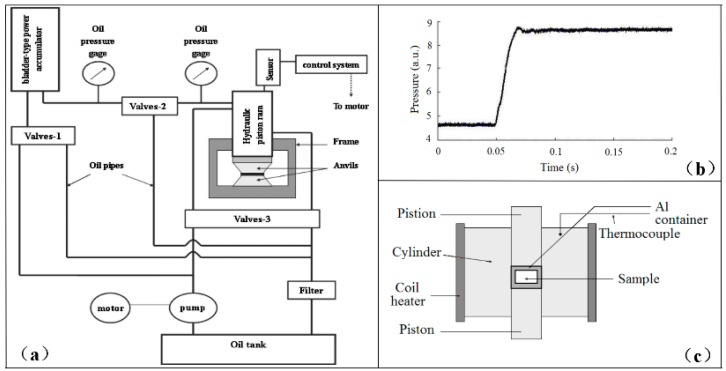
(**a**) Schematic of rapid compression apparatus [10]; (**b**) A typical pressure jump record [10]; (**c**) Sample assembly in the piston–cylinder mold [11].

Rapid compression experiments were conducted on a self-designed apparatus, which was described in detail by Hong et al. [10]. Figure 2b is a typical run of pressure-jump, which shows that the oil pressure in ram rises from 1 MPa to 26.5 MPa in about 20 milliseconds. The high-pressure mold used in the pressure-jump experiments is the piston–cylinder of tungsten carbide [11]. The sample assemblies are shown in Figure 2c. In Figure 2c, the sample was filled in an aluminum container. The sizes of the samples are shown in Table 1. Two diameter-sized piston–cylinder molds, i.e., 20 mm and 26 mm, were used. The pressure was estimated by the diameter of the piston and loaded forces, with the frictions being ignored. The pressure was expected to be independent of the sample size, which was sealed in an aluminum container. A resistance coil heater was used to produce a high temperature. The temperature was measured by a NiCr–NiSi thermocouple. The gradient of temperature between the sample and the detected point has been calibrated. The route of preparing amorphous materials consisted of four steps. Firstly, sample assembly was pre-pressed to eliminate gaps, and then the sample was heated to be molten. Secondly, the melt was rapidly compressed to high pressure through the above mentioned pressure-jump. Thirdly, the pressure was maintained, and the sample was cooled down to room temperature. Finally, the sample was decompressed to ambient pressure.

We performed the rapid compression experiments for PET, PEEK, iPP, HDPE, and sulfur melts. Table 1 shows the pressure and temperature synthesis conditions of different substances [11,12,13,14,15]. The characterizations of the recovered samples were carried out by X-ray diffraction (XRD, X’Pert. PRO. MPD. Philips, using Cu K_α_ radiation) and differential scanning calorimetry (DSC, Netzsch DSC204 Phoenix or STA449C Jupiter, 10 K/min heating rate) [11,12,13,14,15]. 

## 3. Results and Discussion

### 3.1. Solidification of Full Amorphous Phase in PET, PEEK, and Sulfur

#### 3.1.1. PET

**Figure 3:**
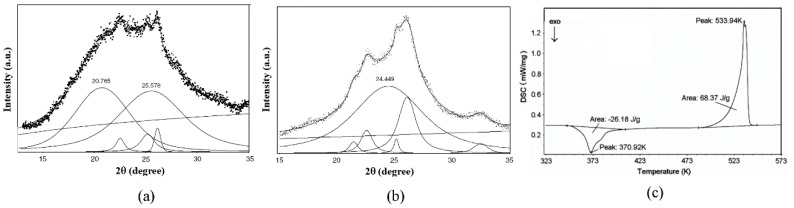
XRD patterns and the peak profile fitting analysis: (**a**) PET sample; (**b**) PET sample after annealing at 403 K for 30 min. (**c**) Differential scanning calorimetry (DSC) curve of the PET sample [12].

PET is a semicrystalline polymer composed of crystalline and amorphous structures. The high-pressure crystallization behavior of PET has been studied intensively [16,17]. It is generally believed that static high pressure could promote the crystallization of PET. The melting point *T*_m_ and crystallization point *T*_c_ of PET increase sharply with increasing pressure; *dT*_m_*/dP* and *dT*_c_*/dP* are about 0.5 K/MPa [18]. This character is propitious for a deep supercooling under high pressure. The rapid compression experiment of PET melt obtained the full amorphous PET sample. In Figure 3a, the XRD pattern of the PET sample demonstrated that the crystalline lines almost disappear, and a smooth diffraction band exists remarkably from 15 to 35 degrees in 2 theta [12]. The smooth diffraction band can be resolved as two components that have peak positions at 25.578 degrees and 20.765 degrees, respectively. The DSC trace of the PET sample in Figure 3c displayed a clear exothermic crystallization at around 371 K and an endothermic melting at 534 K [12]. The final crystallinity is estimated to be 48.84%, of which about 18.70% is contributed from the crystallization of the amorphous phase at 371 K [12]. The latter proportion, 18.70%, is calculated through the enthalpy of the transition in the exothermic valley. In order to study the structure of the crystallization product, the PET sample was heated at 403 K at ambient pressure for 30 min, and then detected by XRD again (Figure 3b). The results showed that after the heat treatment, one amorphous peak remained in the sample, which had the peak position of 24.449 degrees and another amorphous component disappeared along with the increase in the crystalline component. On the basis of these results, it was speculated that there are two kinds of amorphous phases that solidified after the rapid compression process. The amorphous phase residual after heat treatment is relatively stable, which exists in a wide temperature range, even over the melting temperature of the coexisting crystalline. Another is a metastable amorphous phase, which can transform to the crystalline phase at a temperature of about 371 K. This metastable amorphous phase was formed by freezing the parent PET liquid during the rapid compression.

#### 3.1.2. PEEK

**Figure 4:**
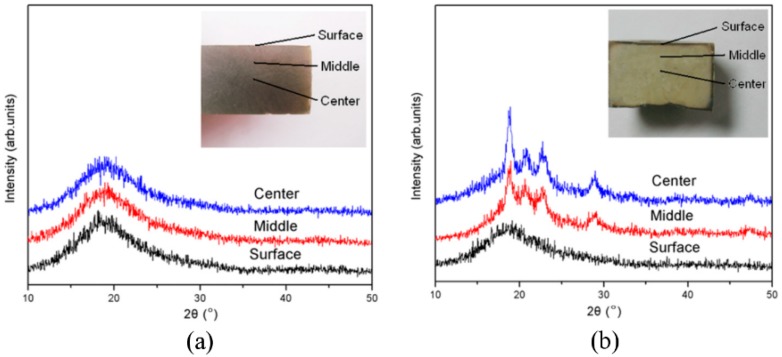
The XRD patterns of PEEK samples along longitudinal section, prepared by (**a**) Rapid compression; and (**b**) Rapid quenching of melt [13].

PEEK is a semicrystalline polymer composed of crystalline and an amorphous phase. Due to its low thermal conductivity, bulk amorphous PEEK has seldom been reported, although the amorphous coating with a thickness of less than 1 mm has been prepared by thermal spray techniques. The rapid compression experiment of PEEK melt obtained fully amorphous PEEK bulk that was 24 mm in diameter and 12 mm in thickness. Figure 4a shows the micro-XRD analysis on the cut surface of the PEEK sample [13]. By comparison, the quenched PEEK sample was prepared by rapidly injecting the melt to ice water, and its micro-XRD analysis is shown in Figure 4b [13]. The three measurement spots are on the surface layer, in the middle area, and at the very center, respectively, as shown in the inset figure. In Figure 4a, the XRD patterns exhibit amorphous character at the three measurement spots, suggesting that fully amorphous phases were formed in the compressed PEEK sample. By contrast, the amorphous phase exists only on the surface layer with a thickness of less than 1 mm in the quenched PEEK sample. It is clear that the size of the amorphous PEEK is as large as 12 mm, which remarkably exceeds the critical size in the quenching process, and is ascribed to the different solidification routes. In the rapidly quenching method, the cooling rate of the sample inner was lower than its surface because of the limit of thermal conductivity. So, only the surface layer solidified to a full amorphous state, and its inner part crystallized. In the rapid compression method, the solidification of melt is realized by changing the pressure instead of the temperature, and the pressure is homogeneously loaded in the whole sample, so the volume of the amorphous phase thus obtained is not limited by the thermal conductivity. 

#### 3.1.3. Sulfur

**Figure 5:**
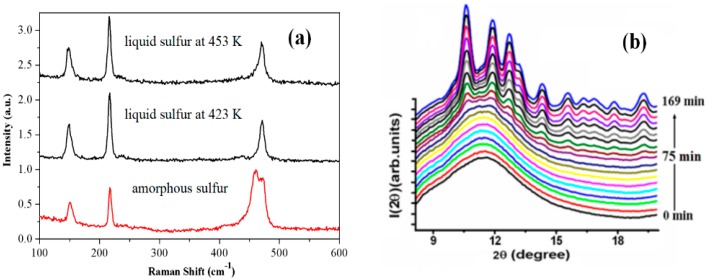

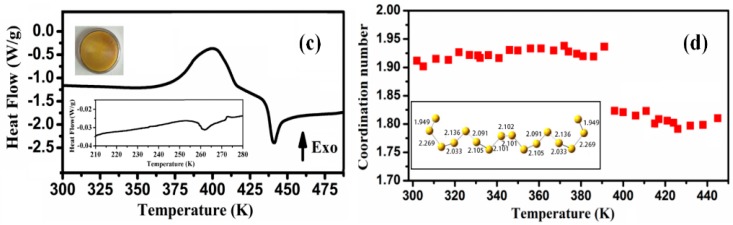
(**a**) Raman spectra of *a*-S at 273 K and after melting at 423 K and 453 K [20]; (**b**) XRD patterns of amorphous sulfur, which is taken at room temperature with a time interval of 10 min [22]; (**c**) Differential scanning calorimetry (DSC) trace of *a*-S under 10 K/min heating rate, the inset heat flow curve is the DSC curve in the range of 210–280 K, and the inset photo is the picture of *a*-S at 291 K [20]; (**d**) Coordination numbers evolution in the first nearest-neighbor shell of *a*-S during heating, the inset figure is the polymeric chain cluster model in *a*-S, and the S-S bond lengths are given in Å [20].

Sulfur is known as a living polymer. Liquid sulfur displays a reversible polymerization transition at around 432 K, which has been interpreted as a kinetically controlled equilibrium reaction between small cyclic monomer molecules and polymeric sulfur molecules [19]. The rapid compression experiment of sulfur melt obtained bulk amorphous sulfur (*a*-S). This *a*-S possesses a much higher content of polymeric chains than its parent liquid [20]. Figure 5a shows the Raman pattern of the *a*-S at 273 K, which was obtained by compressing liquid sulfur at around 453 K. The Raman peaks located at 461 cm^−1^ and 472 cm^−1^ are assigned to the bond-stretching modes of the polymeric chains’ S_μ_ and S_8_ rings, respectively [20]. Their area ratio, *A*^461^/*A*^472^, provides a reasonable fingerprint on the content of the rings and chains [20]. The polymeric chains’ content was about 73 wt%; the majority content is polymeric chains in the *a*-S [20]. This value is obviously higher than that in the parent liquid, which was about 30 wt% at 453 K in Figure 5a [20]. This value is also obviously higher than that in the quenched *a*-S, which was formed via a rapid cooling method e.g., injecting into liquid nitrogen [21]. The quenched *a*-S has almost the same polymer fraction as its parent liquid, since minimal structural changes occur during the quenching process [21]. The difference in the polymer fraction causes an obvious divergence of thermal stability between them, which will be discussed in Section 3.2.2. 

### 3.2. Solidification of Other Polymer Melts

Can all kinds of polymers be solidified to a fully amorphous state? It depends on the non-equilibrium process i.e., rapid compression and rapidly quenching, since it is a beating against crystallization. A higher compression rate or cooling rate is more propitious to prohibit the crystallization. However, for those with a very high tendency of crystallization, it is very difficult to obtain its fully amorphous state. In this work, under similar compression rates with PET, PEEK, and sulfur, the following polymers i.e., iPP and HDPE, cannot be solidified to a fully amorphous state. 

#### 3.2.1. iPP 

**Figure 6:**
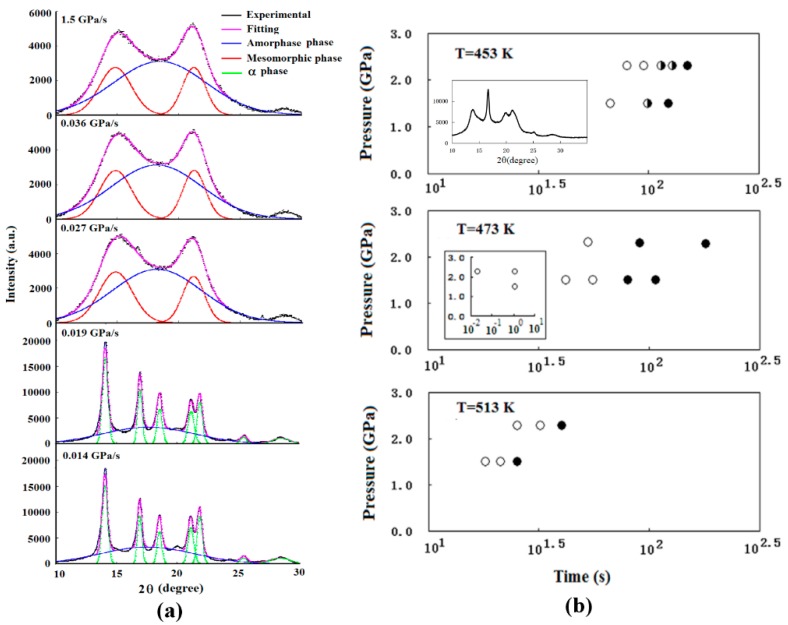
(**a**) The XRD profiles of iPP samples solidified under different compression rates at 473 K [13]. Observed (thick solid lines) and fitting (chain lines) curves, the constituents (dotted lines and thin solid lines) separated by the component analysis are displayed for each profile; (**b**) Structure of iPP samples solidified by compressing at different pressures at different times at 453 K, 473 K, and 513 K from the results of XRD analysis [13]. A full symbol presents the α phase, a void symbol presents the mesomorphic phase, and a half-full symbol presents the coexisting structures of α and mesomorphic phases (as shown in the insert XRD pattern). The insert figure displayed the structure of iPP samples solidified with compression times shorter than 10 s at 473 K.

Isotactic polypropylene (iPP) is a semicrystalline polymer with a simple chemical structure [23,24]. At ambient pressure, the monoclinic α phase is generally the majority phase observed in the isothermal crystallization of melt [23,24]. The melting point of iPP increases sharply with an increase in pressure [18]. This character is propitious for a deep supercooling under high pressure. The rapid compression experiment of iPP melt obtained mesomorphic phase, which is an intermediate state between amorphous and crystal. It was also found that the solidified structure of iPP is strongly dependent on the compression rates. The melt of iPP was solidified by compression at different rates varying from 0.012 GPa/s to 115 GPa/s [15]. Figure 6a shows the XRD patterns of recovered iPP samples after rapid compression at 473 K. It was found that the α phase is solidified at a lower compression rate, and the mesomorphic phase is solidified at a higher compression rate. More experimental results are shown in Figure 6b. Three samples with the coexisting structures of α and the mesomorphic phase are obtained in the third group, in which the temperature 453 K is relatively low and near the melting point at ambient pressure. There exists a very narrow range of compression rates within which the α and mesomorphic phases coexist; for most of the compression conditions, one of the phases largely predominates over the other. Nevertheless, the limit of the compression rate for forming the mesomorphic phase emerges in all of the groups, and so we supposed that there exists a critical compression rate in the pressure-induced solidification process. The mesomorphic phase could be observed when the compression rate is faster than it [15]. 

#### 3.2.2. HDPE 

**Figure 7:**
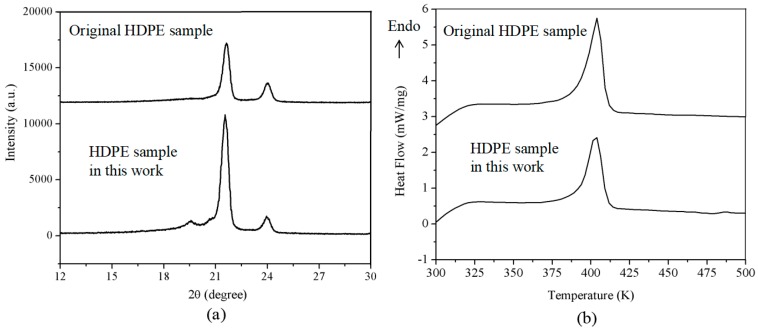
(**a**) The XRD profiles and (**b**) DSC traces of the original HDPE sample and the HDPE sample solidified by rapid compression.

The high-density polyethylene (HDPE) is a typical wondrously crystallized polymer [25]. The rapid compression experiment of HDPE melt was conducted. We compared the XRD and DSC patterns of the HDPE sample thus obtained with the original HDPE sample (Mw = 130,000, Jinshan Petrol. Chem. Co., Shanghai, China). As shown in Figure 7a, the HDPE samples display two strong diffraction peaks at around 2θ = 21.5 degrees and 23.9 degrees, which correspond to (110) and (200) crystal faces, respectively. The HDPE sample solidified by rapid compression has the same crystalline structure as the original HDPE sample. We notice that the peak height ratio *I*_(200)_/*I*_(110)_ is 0.37 and 0.15 for the original HDPE and the HDPE sample in this work, respectively. The relatively intensity decline of the (200) crystal face suggests the reduction of orientation of the molecular chains. In Figure 7b, the DSC traces show that the HDPE samples melt at around 403 K. The crystallinity is calculated by using the equation: X_c_ = Δ*H*_m_/Δ*H*_m_^0^, where Δ*H*_m_^0^ is the equilibrium melting enthalpy, which is 287 J/g, as suggested by Wunderlich [26]. The melting enthalpy Δ*H*_m_ is estimated from the area of the melting peak, and is 225.4 J/g and 168.3 J/g for the original HDPE and HDPE sample in this work, respectively. Consequently, the crystallinity of the HDPE samples is 78.5% and 58.6%, respectively. Although full amorphous HDPE cannot be prepared due to its excellent crystallization ability, it seems that the pressure affected the molecular chain orientation of the crystals and inhabited the crystallization during the rapid compression process. 

### 3.3. Properties of Amorphous PEEK and Amorphous Sulfur

Compared with semicrystalline polymer, full amorphous polymer may possess some unique properties. In addition, polymers usually become more dense under high pressure. So, it is interesting to explore the properties and applications of these polymers. 

#### 3.3.1. Mechanical Properties of Amorphous PEEK

**Figure 8:**
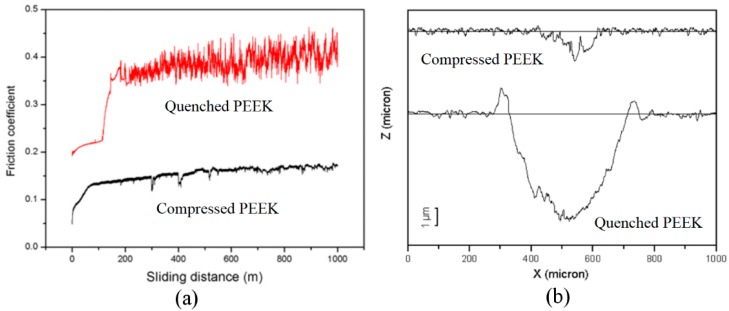
(**a**) The friction coefficient evolution for the samples as a function of sliding distance; (**b**) the profilometry traces of the wear tracks of PEEK samples i.e., the compressed PEEK and the quenched PEEK [13].

We tested the friction coefficient and the wear rate of the two PEEK samples i.e., the compressed PEEK and the quenched PEEK [13]. Figure 8a displayed the friction coefficient curve as a function of sliding distance. The friction coefficient is taken by the mean value of the friction coefficient recorded in the last 500-m sliding distance. The profilometry traces of the wear tracks of the two samples generated during the sliding process are shown in Figure 8b. The bulk amorphous PEEK sample exhibits a very small friction coefficient and an almost inappreciable wear rate, which suggests that it has good friction and wear behavior. The good friction and wear properties were ascribed to the compacted and homogeneous structure with small ordered domains, and also the small average molecular weight [13]. 

#### 3.3.2. Thermal Stability of Amorphous Sulfur

Due to its good intermiscibility with natural and synthetic rubbers, the *a*-S as a vulcanizing agent plays a critical role in the preparation of rubber goods. Compared with the quenched *a*-S, the *a*-S prepared by rapid compression displays extraordinary thermal stability. The quenched *a*-S crystallizes very soon at room temperature [21]. For the *a*-S in this work, it takes about 75 min to start crystallization at room temperature, as shown in Figure 5b [22]. What is interesting is that the *a*-S in this work displays an abnormal exothermic transition to liquid sulfur. When using a heating rate of 10 K/min, the *a*-S can avoid crystallization and transform to liquid sulfur directly, which corresponds to an abnormal exothermic peak at around 396 K, as shown in Figure 5c. The glass transition temperature of *a*-S was around 262 K, as shown in the inset figure of Figure 5c, so the melting of *a*-S is essentially a transition from supercooled liquid to liquid [20]. Raman spectra are taken in the range of 273–473 K to investigate its possible mechanisms [20]. The polymeric chains remained until melting, and turned into S_8_ rings after melting. Figure 5a shows the Raman pattern after melting at 423 K. Synchrotron high-energy XRD were taken in the range of 302–445 K [20]. The average first-neighbor coordination numbers showed an abrupt drop from 1.92 to 1.81 during melting, as shown in Figure 5d. The evolution of the chain length clearly shows that the transition was accompanied by polymeric chains breaking [20]. On the basis of these results, we speculated that the exothermic transition from *a*-S to liquid sulfur likely possesses an additional exothermic contribution stemming from the chain–ring transition. Such an abnormal exothermic phenomenon in *a*-S can be illustrated as the chain–ring transition coupled with the supercooled liquid–liquid transition process [20]. 

## 4. Conclusions

On the basis of the experimental results, it was made clear that the rapid compression of melt is an effective method to inhibit crystallization and prepare full amorphous polymers. Since the solidification process is not limited by thermal conductivity, the size of amorphous polymers thus obtained is expected to exceed the critical size in the rapidly quenching process. It is also prospective that the bulk amorphous polymers thus obtained may possess some unique properties, such as exceptional toughness and high thermal stability. It is a promising way to prepare fully amorphous polymers and explore their properties and applications. In the future, the pressure-induced solidification of melt will be studied in more kinds of polymers.

## Figures and Tables

**Table 1:** The sizes and pressure and temperature synthesis conditions of poly-ethylene-terephthalate (PET), polyether-ether-ketone (PEEK), isotactic polypropylene (iPP), high-density polyethylene (HDPE), and sulfur. The glass transition temperature *T_g_* and crystallization temperature *T_x_* of the recovered samples are also shown.

	Sample Size (Diameter, Height)	Temperature of Melt	Terminal Pressure of Pressure-Jump	Compression Rate	*T_g_*/K	*T_x_*/K
**PET [12]**	22 mm, 4 mm	563 K	2 GPa	100 GPa/s	-	371
**PEEK [13]**	24 mm, 12 mm	633 K	2 GPa	100 GPa/s	413.6	449.1
**Sulfur [11]**	20 mm, 3 mm	423 K	2 GPa	100 GPa/s	262 [14]	-
**iPP [15]**	18 mm, 3 mm	473 K	2.3 GPa	115 GPa/s	-	-
**HDPE**	20 mm 1.8 mm	457 K	2 GPa	100 GPa/s	-	-
